# The Consumption of High-Amylose Rice and its Effect on Postprandial Blood Glucose Levels: A Literature Review

**DOI:** 10.3390/nu16234013

**Published:** 2024-11-23

**Authors:** Jia Li, Kana Yoshimura, Minori Sasaki, Koutatsu Maruyama

**Affiliations:** 1 Laboratory of Community Health and Nutrition, Course of Food Science, Department of Applied Bioresource Science, The United Graduate School of Agriculture Sciences, Ehime University, Matsuyama 790-8566, Japan; 2 Laboratory of Community Health and Nutrition, Special Course of Food and Health Science, Department of Bioscience, Graduate School of Agriculture, Ehime University, Matsuyama 790-8566, Japan

**Keywords:** high-amylose rice, postprandial glucose, review, glycemic index, clinical trials

## Abstract

**Background/Objectives:** Rice is a major staple in the diets of East Asian populations. Numerous meta-analyses have shown an association between high white rice consumption and a higher risk of diabetes. High-amylose rice (varieties with over 25% amylose content) is absorbed more slowly in the gut compared to low-amylose rice, and it results in lower levels of postprandial blood glucose. Various intervention studies have investigated the effects of high-amylose rice consumption on postprandial blood glucose and the glycemic index. The quantity of the research suggests that a comprehensive review of these diverse findings is necessary. **Methods and Results:** We reviewed 17 clinical trials, most of which showed that high-amylose rice ingestion results in lower postprandial blood glucose levels and glycemic index compared to low-amylose rice diets. Although they differed in their sample size, study design, rice type and quantity, and amylose content, most of these studies suggested that there is a reasonable effect of high-amylose rice consumption on postprandial blood glucose. In particular, the effect on blood glucose suppression tended to be related to the amylose content. However, long-term intake studies are still limited and require further investigation. **Conclusions:** In conclusion, high-amylose rice shows promise for blood glucose management.

## 1. Introduction

The International Diabetes Federation (IDF) projected, in its Diabetes Atlas, that over 90% of global diabetes cases are attributed to Type 2 diabetes, with recent estimates indicating that the diabetic population is anticipated to reach 783 million by the year 2045 [1]. In Japan, one of the world’s fastest-aging nations, the estimated number of individuals with glucose intolerance-related health issues, including diabetes, has been continuously increasing over the past three decades [2]. The fundamental approach for managing diabetes encompasses dietary modifications and regular exercise, complemented by pharmacological intervention methods. Despite the diverse array of antihyperglycemic agents at our disposal, over 50% of individuals diagnosed with Type 2 diabetes mellitus fail to attain internationally acknowledged blood glucose targets [3]. The management of hyperglycemia in Type 2 diabetes (2022) consensus report advocates that the incorporation of lifestyle adjustments, including dietary modifications, is a crucial component of the therapeutic regimen [4].

**Figure 1:**
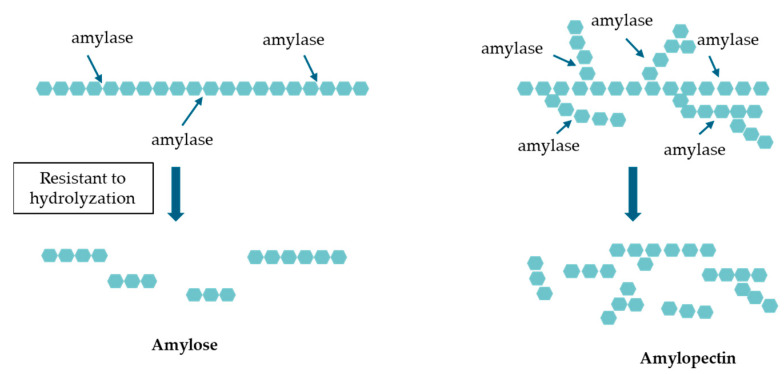
Hydrolysis of amylose and amylopectin. Comparison of the amylose and amylopectin digestion mechanisms. Amylose’s linear structure resists hydrolysis via amylase, slowing glucose release while amylopectin’s branched structure allows for faster enzymatic digestion, leading to rapid blood glucose elevation.

Rice is a staple food in East Asian diets, and numerous associations between diabetes and dietary habits have been documented, with particular emphasis being placed on the link between the consumption of white rice and the risk of developing diabetes [5,6,7]. Both domestic and international studies, supported by multiple meta-analyses, have demonstrated a notable association between elevated white rice consumption and an augmented risk of diabetes [8,9,10]. However, there are also low-glycemic index (GI) foods, such as brown rice and high-amylose rice (varieties with an amylose content exceeding 25%), which are digested slowly in the intestines and do not raise postprandial blood glucose levels [11,12]. As shown in Figure 1, unlike the highly branched structure of amylopectin, the linear structure of amylose makes it resistant to hydrolysis via α-amylase, resulting in a slower breakdown rate; this characteristic classifies it as type 2 resistant starch, which contributes to its ability to moderate blood glucose response [13,14].

Numerous studies have investigated the mitigation of elevated postprandial blood glucose levels and GI regarding high-amylose rice consumption [15,16,17,18]. However, these studies have varied in their sample size, study design, rice type and volume, and amylose content. To draw meaningful conclusions regarding the impact of high-amylose rice on postprandial blood glucose levels, there is a need to systematically organize these studies’ findings. To date, a comprehensive review of the results of previous studies has been lacking. Consequently, the present study employed both national and international literature databases to conduct a review of the data associated with the consumption of high-amylose rice and its impact on postprandial blood glucose levels.

## 2. Materials and Methods

### 2.1. Search Methods for Articles

This review followed the PRISMA statement guidelines [19]. Literature related to high-amylose rice and its impact on postprandial blood glucose levels was gathered. The literature search included articles published between January 1984 and April 2024, and it was conducted using PubMed, Google Scholar, and Ichushi Web (Japanese), which is developed and maintained by the NPO Central Journal of Medical Science. The keywords used for the search were “high amylose rice”, “glucose”, and “glycemic index”.

### 2.2. Screening and Selection of Articles

Articles were screened using the following inclusion criteria: (1) the study design was a clinical trial; (2) the exposure was the consumption of high-amylose rice; and (3) the outcomes included changes in postprandial blood glucose levels and the GI. The following were excluded: (1) studies involving animal subjects; (2) processed products containing high-amylose rice (e.g., cakes, porridge); and (3) duplicate publications, books, conference abstracts, and similar materials.

### 2.3. Article Information Summary

From each article, a multitude of information was extracted and summarized, including, but not limited to, the study design, country/region where the study was conducted, participant characteristics (age, gender, disease status, and sample size), study methods (intervention, exposure, and control), outcome assessment/results (mean postprandial area under the curve (AUC)/incremental area under the curve (IAUC) values, GI, and glycemic load), and rice amylose content (Table 1 and Table 2).

## 3. Results

### 3.1. Literature Search

**Figure 2:**
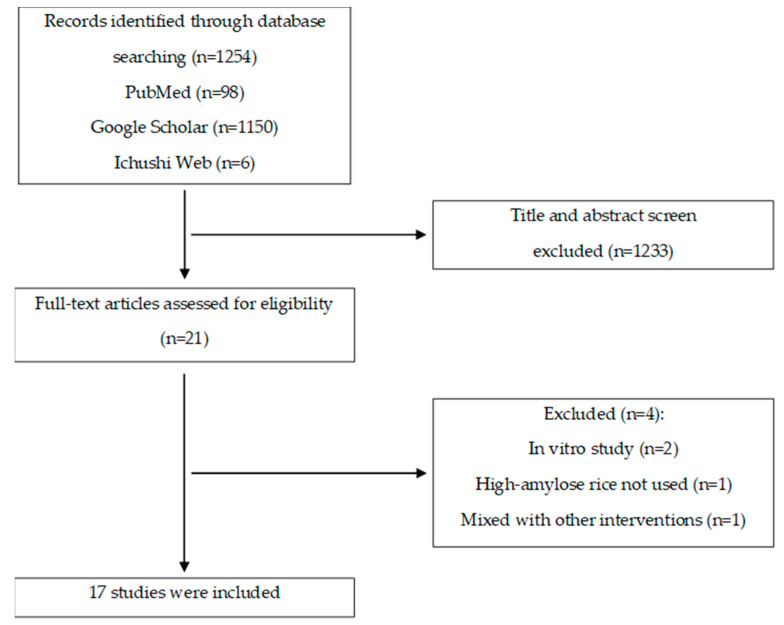
Flow diagram of the study selection processes.

The literature search yielded a total of 1254 articles, comprising 98 from PubMed, 1150 from Google Scholar, and 6 from Ichushi Web. Following a thorough screening process involving titles, abstracts, and related criteria, 21 articles (i.e., 21 studies) were selected for a comprehensive full-text review. Subsequently, a total of 17 articles were used after 4 articles were excluded that did not align with our predefined inclusion criteria (pertaining to study design, intervention, subjects, or outcomes) (Figure 2).

### 3.2. Study Characteristics

The 17 selected studies [15,16,17,18,20,21,22,23,24,25,26,27,28,29,30,31,32] originated from various countries (as shown in Table 1 and Table 2): 7 were from Japan, 3 from the United States, 2 from Bangladesh, and 1 each from Malaysia, Denmark, Australia, the Philippines, and Sri Lanka. Regarding the individuals included in the respective studies, 15 involved healthy adults, while 2 involved individuals with Type 2 diabetes. A total of 16 of the studies compared postprandial glucose AUC or IAUC, and 10 documented GI values.

### 3.3. Effect of High-Amylose Rice Consumption on Glucose AUC or IAUC

In the studies comparing the AUC or IAUC, 9 out of 14 reported significantly lower postprandial glucose AUC or IAUC in individuals after consuming high-amylose rice compared to those who consumed control rice (low-to-middle-amylose rice) (Table 1) [15,16,20,21,24,25,26,27,28]. Two of these studies involved diabetic individuals and demonstrated the effectiveness of high-amylose rice in inhibiting blood glucose elevation [20,21]. Moreover, among the seven studies featuring an amylose content exceeding 27%, six studies showed a significantly lower glucose AUC or IAUC in individuals after consuming high-amylose rice [15,20,21,24,26,27]. Additionally, among the seven studies featuring relatively low amylose content (<27%), four highlighted the insignificant effect that high-amylose rice consumption had on postprandial glucose levels [17,18,31,32].

Regarding studies using glucose as the reference food, a total of five studies were selected. Among these studies, two reported numerical values. The median IAUC level observed in individuals after consuming glucose was 4325 (range: 2955–5695) mg×min/dL while the median IAUC for those who consumed high-amylose rice was 2658 (range: 1969–2800) mg×min/dL [22,31]. Among the five studies reporting significant differences due to diet, compared to those who consumed normal rice where the median IAUC observed was 3518 (range: 2502–4248) mg×min/dL while those whose diet focused on high-amylose rice displayed a median of 1767 (range: 1389–3384) mg×min/dL [16,24,26,27]. The median AUC for individuals who consumed normal rice was 8240 (range: 3519–12,960) mg×min/dL, and it was 2419 (range: 2170–11,640) mg×min/dL for those who consumed high-amylose rice [15,16].

### 3.4. Effect of High-Amylose Rice Consumption on the GI

A total of ten of the selected papers focused on documenting the GI values, as shown in Table 1 and Table 2. Among the five studies calculating GI based on glucose consumption, two reported that high-amylose rice, when consumed, aids in lowering the GI (i.e., “low GI food”) [22,24], whereas two classified it as a “medium GI food” [23,30]. In these ten studies, the median GI value calculated with glucose as the reference food was 61 (range: 43–86), while that calculated with normal rice or white bread as the reference food was 50 (range: 45–86).

## 4. Discussion

In this study, 17 clinical trials were reviewed, with sample sizes ranging from 4 to 33 participants. Most of the results showed that, compared to those consuming normal rice, individuals consuming high-amylose rice exhibited lower postprandial blood glucose levels and GI. Regarding the GI, most of the studies indicated that high-amylose rice belongs in the category of medium-to-low GI foods. Compared to normal white rice or white bread, high-amylose rice consumption led to a lower GI in participants. Although the results of AUC and IAUC for postprandial blood glucose levels after consuming high-amylose rice were not significant in individual studies, a significant reduction in the initial blood glucose levels and peak blood glucose levels was observed, and, in some studies, the overall insulin secretion responses were significantly lower [17,32]. Overall, these data indicate the potential for high-amylose rice to delay glucose digestion and absorption.

Potential reasons for the inconsistency in these findings may be the amylose content levels in experimental diets and the ethnicity of participating individuals. In our review, the effects on the inhibition of postprandial blood glucose elevation were more pronounced in the studies where the content of amylose in rice exceeded 27% [15,20,21,22,23,24,26,27]. In particular, as observed in the studies by Fatema K. et al. and Pathiraje P. et al., among various high-amylose rice types, the rice with the lowest amylose content (24.5%) had the highest GI (GI = 73). However, when the amylose content exceeded 27%, the GI was consistently below 70, which suggests a correlation between the effectiveness of inhibiting the blood glucose elevation and amylose content [22,23]. Previous studies found that the hydrolysis rate of starch in high-amylose rice is significantly lower than that in normal rice [33]. Moreover, as the amylose content increases, the hydrolysis rate slows down, resulting in reduced digestion and absorption rates [34]. This may explain why higher amylose content is associated with providing better glycemic control. The majority of participants were Asian in the studies and no significant differences were shown. Therefore, the results may be due to ethnic differences as Asians tend to have lower insulin sensitivity and higher insulin resistance than Caucasians, according to relevant research [35]. Additionally, both studies focusing on Type 2 diabetes demonstrated a lower increase in postprandial blood glucose levels compared to individuals with normal rice diets [20,21]. This suggests that high-amylose rice may have a more marked (and beneficial) effect in diabetic patients. Consistent with previous reviews, the higher proportion of amylose could be associated with lower postprandial blood glucose [36]. Moreover, the present review further explores this relationship and suggests that an amylose content of approximately 27% may play a significant role in influencing postprandial blood glucose. Unlike earlier reviews that primarily provided a general evaluation of various rice types or starchy foods, this review discusses whether high-amylose rice specifically contributes to the suppression of postprandial hyperglycemia [36,37].

It must be noted that this study had some limitations. Firstly, a limited number of published papers fit the search criteria, and the rice cooking methods varied across different studies. For instance, in the Goddard M.S. et al. and Juliano B.O. et al. studies, microwave-cooked rice was utilized [17,29], while the participants in the studies of Zenel A.M. et al. and Saito Y. et al. consumed refrigerated and reheated rice [15,26]. Previous research indicates that different cooking and processing methods can influence the digestibility and postprandial glycemic responses of various rice varieties [38,39,40]. Additionally, studies by Yamaguchi T. et al. and Mori H. et al. used the same high-amylose rice as the test food, but differences such as water-to-rice ratios in the processing methods used led to varying outcomes [25,32]. Furthermore, the literature selection process in the present review was constrained by the keywords and the types of test foods. Studies from previous related reviews, such as those focusing on resistant starch rice, were excluded not only due to differences in keywords but also because they did not clearly identify high-amylose rice as the main subject [41]. Similarly, studies involving meals that combined high-amylose rice with other ingredients, such as amylomaize starch, were excluded as they did not solely focus on high-amylose rice [42]. Secondly, factors such as the participants’ characteristics, particularly whether they are undergoing diabetic treatment or not, warrant further investigation.

Multiple studies have indicated the beneficial effects of high-amylose rice in inhibiting postprandial blood glucose elevation [15,16,20,21,22,23,24,25,26,27,28], with two studies showing particular effectiveness in diabetic patients [20,21], thus suggesting its potential value in diabetic dietary management. However, the data on this topic remain limited, and further research is required. Additionally, the unique texture of high-amylose rice and the culinary preferences may limit consistent rice consumption by certain individuals.

## 5. Conclusions

Overall, high-amylose rice holds promise for blood sugar management. In conclusion, a tendency toward lower postprandial blood glucose levels was observed as a result of high-amylose-focused diets, suggesting that high-amylose rice may have a beneficial effect on postprandial hyperglycemia.

## Figures and Tables

**Table 1:** Description of studies showing significant effects of high-amylose rice on the AUC/IAUC of the postprandial glucose levels.

Author, Year	Design	Country	Number of Participants (Male/Female)	Mean or Range of Participants’ Age (Years)	Intervention *	Control	Outcome	AUC (mg×min/dL)	IAUC (mg×min/dL)	Significant Difference (*p*-Value)	GI	
Larsen H.N. et al., 1996 [20]	Cross-over study	Denmark	7/5 (diabetic)	45–76	50 g available carbohydrate as a standard for high-amylose rice (a: 28, b: 28, and c: 27%) and low-amylose rice (12%)	50 g available carbohydrate as a standard for white bread	Postprandial blood glucose at 0, 15, 30, 45, 60, 90, 120, 150, and 180 min	N/A	High-amylose rice a: 7398, b: 7038, and c: 6498, low-amylose rice 10188	High-amylose rice was significantly lower (*p* < 0.01)	Using white bread as the reference (GI = 100), the high-amylose rice GIs were a: 53, b: 50, and c: 47	The high-amylose rice GI was significantly lower than the low-amylose rice GI (*p* < 0.01)
Parvin S. et al., 2009 [21]	Cross-over study	Bangladesh	8/9 (diabetic)	42 ± 5	50 g available carbohydrate as a standard for high-amylose rice (a: 29, b: 29, and c: 27%) and low-amylose rice (13%)	50 g available carbohydrate as a standard for white bread	Postprandial blood glucose at 0, 15, 30, 45, 60, 90, 120, 150, and 180 min	N/A	High-amylose rice a: 7398, b: 7038, and c: 6498, low-amylose rice 10188	High-amylose rice was significantly lower (*p* < 0.05)	Using white bread as the reference (GI = 100), the high-amylose rice GIs were a: 52, b: 50, and c: 47	The high-amylose rice GI was significantly lower than the low-amylose rice GI (*p* < 0.01)
Fatema K. et al., 2010 [22]	Single-blind, cross-over study	Bangladesh	5/5	28.6 ± 5.7	180, 150, and 175 g of high-amylose rice (a: 27, b: 29.4, and c: 27.2%)	50 g of glucose	Postprandial blood glucose at 0, 30, 60, 90, and 120 min	N/A	High-amylose rice a: 2786, b: 2800, and c: 1969, glucose 5695	High-amylose rice was significantly lower for GLU	Using GLU as the reference (GI = 100), the high-amylose rice GIs were a: 54.5, b: 50.3, and c: 43.1	Low GI
Pathiraje P. et al., 2010 [23]	Randomized controlled trial	Sri Lanka	10 (In total)	25–45	195, 162, 150, 143, 181, 191, and 173 g of high-amylose rice (a: 27.1, b: 27.0, c: 29.0, d: 24.5, e: 29.5, f: 29.0, and g: 27.7%) and 149 g middle-amylose rice (21.5%)	50 g of glucose	Postprandial blood glucose at 0, 15, 30, 45, 60, 90, and 120 min	N/A	N/A	High-amylose rice was significantly lower for GLU	Using GLU as the reference (GI = 100), the high-amylose rice GIs were a: 61, b: 67, c: 67, d: 73, e: 60, f: 57, and g: 62	The high-amylose rice GI was significantly lower than the middle-amylose rice GI (*p* < 0.01)
Unno R. et al., 2012 [16]	Single-blind, cross-over study (sorted by weight)	Japan	20/0	25 ± 5	173 g of high-amylose rice (25%)	159 g of normal rice	Postprandial blood glucose at 0, 30, 45, 60, and 120 min	High-amylose rice 11640, normal rice 12960	High-amylose rice 1680, normal rice 3120	High-amylose rice was significantly lower (*p* < 0.001)	Using normal rice as the reference (GI = 100), the high-amylose rice GI was 50	
Trinidad T.P. et al., 2013 [24]	Cross-over study	Philippines	9–10 (in total)	27–55	50 g available carbohydrate as a standard for high-amylose rice (27%) and various low-to-middle-amylose rice (0.6–22.9%)	50 g of glucose	Postprandial blood glucose at 0, 15, 30, 45, 60, 90, and 120 min	N/A	High-amylose rice 3384, low-to-middle amylose rice (3816–5040)	High-amylose rice was significantly lower (*p* < 0.05)	Using GLU as the reference (GI = 100), the high-amylose rice GI was 50	Low GI
Zenel A.M. et al., 2015 [15]	Randomized, single-blind cross-over study	USA	9/9	21–37	1/2 cup US (120 mL) of raw high-amylose rice (a: 29 and b: 30.3%)	1/2 cup US (120 mL) of raw normal rice	Postprandial blood glucose at 0, 15, 30, 45, 60, 90, and 120 min	High-amylose rice a: 2170 and b: 2419, normal rice 3519	N/A	High-amylose rice was significantly lower (*p* = 0.006)	N/A	
Yamaguchi T. et al., 2019 [25]	Single-blind cross-over study	Japan	6/6	21.3 ± 1.1	50 g available carbohydrate as a standard for high-amylose rice (26.3%)	50 g available carbohydrate as a standard for normal rice	Postprandial blood glucose at 0, 15, 30, 45, 60, 90, and 120 min	N/A	N/A	High-amylose rice was significantly lower (*p* < 0.05)	N/A	
Saito Y. et al., 2020 [26]	Randomized, single-blind cross-over study	Japan	17/2	24.1 ± 5.9	175 g of high-amylose rice (45%)	160 g of normal rice	Postprandial blood glucose at 0, 30, 45, 60, and 120 min	N/A	High-amylose rice 1854, normal rice 2502	High-amylose rice was significantly lower (*p* = 0.021)	N/A	
Tamura A. et al., 2021 [27]	Randomized, double-blind cross-over study	Japan	0/4	21.3 ± 0.5	50 g available carbohydrate as a standard for high-amylose rice (33%)	50 g available carbohydrate as a standard for normal rice	Postprandial blood glucose at 0, 30, 60, 90, and 120 min	N/A	High-amylose rice 1389, normal rice 3219	High-amylose rice significantly lower (*p* < 0.05)	Using normal rice as the reference (GI = 100), the high-amylose rice GI was 45	The high-amylose rice GI was significantly lower than the normal rice GI (*p* < 0.05)
Yamaguchi T. et al., 2021 [28]	Single-blind, cross-over study	Japan	5/5	21.3 ± 1.3	50 g available carbohydrate as a standard for high-amylose rice (26.3%)	50 g available carbohydrate as a standard for normal rice	Postprandial blood glucose at 0, 15, 30, 45, 60, 90, and 120 min	N/A	N/A	High-amylose rice was significantly lower (*p* < 0.05)	N/A	

* The content of amylose (%) is shown in the parentheses.

**Table 2:** Description of studies showing no significant effects or unmeasured outcomes of high-amylose rice on the AUC/IAUC of the postprandial glucose levels.

Author, Year	Design	Country	Number of Participants (Male/Female)	Mean or Range of Participants’ Age (Years)	Intervention *	Control	Outcome	AUC (mg×min/dL)	IAUC (mg×min/dL)	Significant Difference	GI	
Goddard M.S. et al., 1984 [17]	Cross-over study	USA	17/16	27–81	50 g available carbohydrate as a standard for high-amylose rice (23–25%), middle-amylose rice (14–17%), and low-amylose rice (0%)	50 g of glucose	Postprandial blood glucose at 30, 60, 120, and 180 min	N/A	N/A	High-amylose rice was significantly lower for GLU but not significantly different to low-to-middle-amylose rice	N/A	
Juliano B.O. et al., 1986 [29]	Cross-over study	USA	16 (in total)	N/A	50 g available carbohydrate as a standard for high-amylose rice (28.4%)	50 g available carbohydrate as a standard for normal rice	Postprandial blood glucose at 0, 30, 60, 120, and 180 min	N/A	N/A	No significant difference (*p >* 0.05)	N/A	
Miller J.B. et al., 1992 [30]	Cross-over study	Australia	8 (in total)	19–36	64.5 and 67.5 g of raw high-amylose rice (28%)	50 g available carbohydrate as a standard for white bread	Postprandial blood glucose at 0, 15, 30, 60, 90, and 120 min	N/A	N/A	N/A	Using GLU as the reference (GI = 100), the high-amylose rice GI was 64	
Karupaiah T. et al., 2011 [31]	Cross-over study	Malaysia	6/4	22.6 ± 1.1	50 g available carbohydrate as a standard for brown rice (19%), the polished grain of brown rice (23%), and white-polished rice (26.5%)	50 g of glucose	Postprandial blood glucose at 0, 15, 30, 45, 60, 90, 120, and 180 min	N/A	High-amylose rice 2530, brown rice 1514, polished grains of brown rice 2336, glucose 2955	No significant difference was found between the test rice types (*p* > 0.05)	Using GLU as the reference (GI = 100), brown rice 51, white-polished rice 86, polished grain of brown rice 79	
Mori H. et al., 2018 [32]	Randomized, double-blind cross-over study	Japan	8/8	22.3 ± 1.3	50 g available carbohydrate as a standard for high-amylose rice (26.3%)	50 g available carbohydrate as a standard for normal rice	Postprandial blood glucose at 0, 15, 30, 45, 60, 90, and 120 min	High-amylose rice 964, normal rice 1679	N/A	No significant difference (*p* = 0.138)	N/A	
Maruyama K. et al., 2019 [18]	Non-randomized, single-blind study	Japan	8/0	37.1 ± 9.8	139 g of high-amylose rice (24.9%)	147 g of normal rice	Postprandial blood glucose at 0, 15, 30, 45, 60, 90, and 120 min	High-amylose rice 4015.3, normal rice 4872.5	N/A	No significant difference (*p* = 0.13)	Using normal rice as the reference (GI = 100), the high-amylose rice GI was 86.2	

* The content of amylose (%) is shown in the parentheses.

## Data Availability

The data presented in this study are derived from publicly available resources. These data were obtained from the following public domain sources: PubMed, available at https://pubmed.ncbi.nlm.nih.gov. Google Scholar, available at https://scholar.google.com. Ichushi Web, available at https://search.jamas.or.jp/. References to the articles retrieved from these platforms are provided in the manuscript.
